# Contextual and Temporal Constraints for Attentional Capture: Commentary on Theeuwes’ 2023 Review “The Attentional Capture Debate: When Can We Avoid Salient Distractors and when Not?”

**DOI:** 10.5334/joc.274

**Published:** 2023-07-06

**Authors:** MaryAnn P. Noonan, Viola S. Störmer

**Affiliations:** 1 Department of Psychology, University of York, Heslington, York, UK; 2 Department of Experimental Psychology, University of Oxford, South Parks Road, Oxford, UK; 3 Department of Psychological and Brain Sciences, Dartmouth College, USA

**Keywords:** Cognitive Control, Attention, Implicit learning

## Abstract

Salient distractors demand our attention. Their salience, derived from intensity, relative contrast or learned relevance, captures our limited information capacity. This is typically an adaptive response as salient stimuli may require an immediate change in behaviour. However, sometimes apparent salient distractors do not capture attention. Theeuwes, in his recent commentary, has proposed certain boundary conditions of the visual scene that result in one of two search modes, serial or parallel, that determine whether we can avoid salient distractors or not. Here, we argue that a more complete theory should consider the temporal and contextual factors that influence the very salience of the distractor itself.

## Introduction

There is a longstanding and ongoing debate about whether physically salient stimuli capture attention automatically or whether capture can be avoided by suppressing distractors via top-down mechanisms. The current commentary by Theeuwes critically adds to this debate by carefully carving out the task conditions in which distractors capture attention or not. In this commentary we propose that the boundary conditions that determine stimulus saliency must accommodate temporal and contextual factors such as experience or current cognitive state.

## How do we determine the boundary conditions?

Theeuwes argues that saliency of the target and the distractor, determined by the number of items in the visual scene as well as global heterogeneity of the visual environment (Duncan and Humphreys 1989; Nothdurft 1993), result in one of two attentional search strategies, parallel or serial search, being implemented to identify the target. The adoption of these two search modes will determine whether the distractor captures attention or not. This theory elegantly builds on the signal suppression hypothesis (Gaspelin & Luck 2018) and relates neatly to the earlier feature integration theory (Treisman and Gelade 1980; Treisman and Sato 1990) by attempting to construct the boundary conditions under which these two mechanisms are engaged by the brain. However, we challenge this theory to directly examine the processes that establish the boundary conditions themselves. What determines stimulus saliency? Are these boundary conditions static or can they change with external task demands or internal cognitive constraints or experiences, and if so, how can we measure this? We hope in exploring these questions we can begin to consider these conditions as dynamic and adaptive gradients.

## What determines saliency?

Theeuwes argues that attentional capture occurs only if the target is salient and the distractor is even more salient than the target, as this determines whether participants employ ‘parallel’ or ‘serial’ search strategies. During serial search, capture can be avoided because of the relatively small attentional window that would prevent processing salient distractors. In contrast, during parallel search, the attentional window is relatively large and thus can include potential distractors that then capture attention.

**Figure 1:**
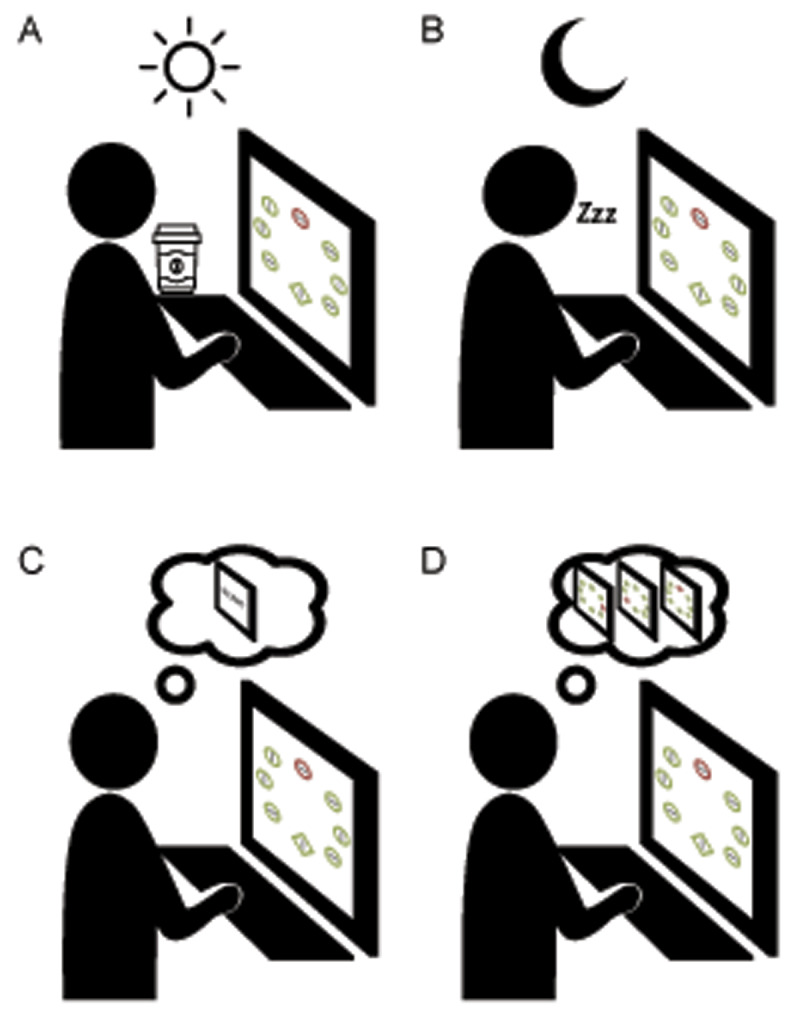
**Attentional capture under different contextual and temporal states. [A, B].** Differences in neurochemical (coffee cup, e.g.,) or cognitive (sleepy “Zzz”, e.g.) states between or within subjects performing an additional singleton paradigm may affect attentional capture differentially, even when experimental factors are identical. **[C, D].** Differences in experience with distractor features across a test session, from the first trial in a session (START screen) to a number of trials later (memories of past trials) may affect distractor influence on search mode. (Icons adapted from the “Noun Project” artists Umehara, T., Made, and Coquet, A.).

We question this dichotomous and rather static view of parallel vs. serial search modes where distractors either “capture” or do “not capture” attention due a particular display type. Rather, search, and therefore attentional capture, could be a dynamic process, within a trial and across trials. If we consider that stimulus saliency is unlikely to be a fixed constant, searching for an item may actually include a mixture of search modes, with one display being more capture-like in one situation or moment in time, but less capture-like in another, or for another person, at a different time of day, etc. (Figure 1). Thus, there is no “magic number” of items in a display that will either trigger capture or not, or a fixed amount of item heterogeneity that will always avoid capture by any distractor. It seems critical to include these more continuous aspects of search and what the boundary conditions are across different situations or prior experiences. Indeed, there may already be room for these ideas within Theeuwes’ theory as searches described as “partially serial” are noted. But what does “partially serial” search look like and what contexts does it manifest in? Some recent accounts propose a more continuous process by which saliency on each trial is computed through accumulating a contrast signal by actively comparing the target template in working memory to all items on the display (Buetti, Cronin et al. 2016). Here, the speed of ‘parallel’ search is determined by the contrast between the target template and each item, and thus depends on target-distractor similarity and the number of items on the display (Lleras, Wang et al. 2020), which is distinct from more classic accounts where the time to compute a saliency map is usually seen as constant and independent of display size. Models like this may help formalize and better quantify saliency and could include some of the more dynamic and continuous aspects of search behavior within the same trial.

## Where does the knowledge during serial search come from?

Theeuwes argues that when subjects engage in a feature search mode (i.e. serial search) search is “*under top-down control guided by knowledge regarding the target (guided search) and knowledge about what is not the target (signal suppression, feature inhibition)”*. Having questioned the nature of saliency, we also ask where does this knowledge come from? If targets or distractors have been explicitly cued, knowledge can be represented actively in working memory. Given that working memory contents automatically boost processing of matching sensory input (Soto, Heinke et al. 2005), even when detrimental to the task (Soto & Humphreys 2009), we arrive at the classic “white bear” paradox (Wegner 1989). This may lead to a form of reactive ignoring, where distractors are initially enhanced, but then subsequently discarded (‘search-and-destroy’; (Moher & Egeth 2012, see also Addleman & Störmer 2022). By contrast, if knowledge is implicitly acquired through repeated experience and its presence becomes predictable it can ultimately be ignored and even suppressed relative to non-distractor baseline measures (Cunningham & Egeth 2016; Leber, Gwinn et al. 2016; Noonan, Adamian et al. 2016; Stilwell & Vecera 2019; Wang, van Driel et al. 2019; Won and Geng 2020), potentially through expectation-based suppression or predictive coding mechanisms (Friston 2010; Slagter & van Moorselaar 2021). This research illustrates the importance of examining performance across time instead of simply averaging across all trials or sessions (van Moorselaar & Slagter 2019; van Moorselaar & Slagter 2020; Addleman & Störmer 2022; Noonan, Von Lautz et al. 2022). Further, novel neural decoding techniques which measure the strength of stimulus representations enable us to directly assess how neural populations tuned to distractor features change with experience of the task and the stimuli. These methods could also be leveraged to establish whether a stimulus is effectively excluded from processing (ignored) or suppressed, an issue that has recently been explored in Wöstmann et al. (2022). Future work using neuroimaging techniques and appropriate baselines will add to our understanding of how knowledge derived through repeated experience with targets and distractors affects the saliency of these stimuli and critically their impact on attentional capture.

## How do these boundary conditions translate to behavior in the real world?

The real world is much more complex and dynamic relative to lab-based tasks upon which most cognitive theories are constructed. Determining the precise boundary conditions of set size and inter-item heterogeneity of a display screen in the lab is important but we must remember that looking out our windows at the end of a long day presents us with a much more unconstrained problem. Thus, considering at least some aspects of the complexity of the real world (such as these temporal and contextual factors) is critical when conceiving of a new model of attentional capture. Indeed, many of the challenges of attentional control in lab studies may be counteracted by reliance on other mechanisms (as seen in visual search in real-world scenes, attention orienting and working memory such as long-term memory, statistical learning and reinforcement learning; (Summerfield, Lepsien et al. 2006; Collins & Frank 2012; Le-Hoa Võ & Wolfe 2015; Geng, DiQuattro et al. 2017; Yu and Geng 2019), which may provide complementary information to improve noisy searches. Complete theories of attentional capture should acknowledge the influence or intersection of these other mechanisms on dynamic saliency maps and determine how they ultimately change how we deal with distraction. This is important as the interaction of these cognitive systems may lead to tell-tale errors when these regularities cannot be relied upon. As such careful experimental design will be useful in describing populations with apparent attentional control deficits, as these other factors might provide alternative or complementary explanations for their cognitive differences.

## Concluding remarks

Theeuwes argues that the primary determining factor of whether a distractor captures attention or not depends on the saliency of both the search target and distractor. Here we argue that heterogeneity and search set size are likely not the only determining factors of stimulus saliency. Instead, temporal and contextual factors that vary on a trial-wise basis or differ across individuals must be accommodated into a complete model. We suggest that future studies examining the influence of these factors on attentional capture will likely benefit from new experimental paradigms that more clearly distinguish distractor and target processing, new analysis methods that examine trial-wise differences, and neuroimaging techniques that estimate the strength of neural populations tuned to distractor features.
